# Patrilineal Background of Esophageal Cancer and Gastric Cardia Cancer Patients in a Chaoshan High-Risk Area in China

**DOI:** 10.1371/journal.pone.0081670

**Published:** 2013-12-10

**Authors:** Shuhui Liu, Bo Huang, Haihua Huang, Xiaoyun Li, Guangcan Chen, Guohong Zhang, Wengting Lin, Dan Guo, Jie Wang, Zefeng Yu, Xi Liu, Min Su

**Affiliations:** 1 Institute of Clinical Pathology & Department of Pathology, Guangdong Provincial Key Laboratory of Infectious Diseases and Molecular Immunopathology, Shantou University Medical College, Shantou, Guangdong, China; 2 Department of Pathology, the Second Affiliated Hospital of Shantou University Medical College, Shantou, Guangdong, China; 3 Department of General Surgery, the First Affiliated Hospital of Shantou University Medical College, Shantou, Guangdong, China; Kagoshima University Graduate School of Medical and Dental Sciences, Japan

## Abstract

The Taihang Mountain range of north-central China, the Southern region area of Fujian province, and the Chaoshan plain of Guangdong province are 3 major regions in China well known for their high incidence of esophageal cancer (EC). These areas also exhibit high incidences of gastric cardia cancer (GCC). The ancestors of the Chaoshanese, now the major inhabitants in the Chaoshan plain, were from north-central China. We hypothesized that EC and GCC patients in Chaoshan areas share a common ancestry with Taihang Mountain patients. We analyzed 16 East Asian-specific Y-chromosome biallelic markers (single nucleotide polymorphisms; Y-SNPs) and 6 Y-chromosome short tandem repeat (Y-STR) loci in 72 EC and 48 GCC patients from Chaoshan and 49 EC and 63 GCC patients from the Taihang Mountain range. We also compared data for 32 Chaoshan Hakka people and 24 members of the aboriginal She minority who live near the Chaoshan area. Analysis was by frequency distribution and principal component, correlation and hierarchical cluster analysis of Y-SNP. Chaoshan patients were closely related to Taihang Mountain patients, even though they are geographically distant. Y-STR analysis revealed that the 4 patient groups were more closely related with each other than with other groups. Network analysis of the haplogroup O3a3c1-M117 showed a high degree of patient-specific substructure. We suggest that EC and GCC patients from these 2 areas share a similar patrilineal genetic background, which may play an important role in the genetic factor of EC and GCC in these populations.

## Introduction

Esophageal cancer (EC) is one of the most common fatal cancers worldwide. China has geographical “hot spots” of high EC incidence. A well-known region with high risk of EC in China is the Taihang Mountain area between Henan, Hebei, and Shanxi provinces in north-central China, the famous “Asian EC belt” ranging from the Caucasian mountains, across northern Iran, all the way to northern China [1]. As well, the incidence of gastric cardia cancer (GCC) is high in the belt. For example, the world standardized incidence of EC and GCC in Linxian, Henan province, was 81.96/100,000 people and 31.04/100,000, respectively between 1983 and 2002 [2], [3]. The Chaoshan area in southern China is another EC high-risk area. The age-standardized incidence rates in Nanao island for EC and GCC were 74.47/100,000 and 34.81/100,000, respectively, between 1995 and 2004 [4].

The geographic features of south-littoral Chaoshan and north-central Taihang Mountain area are distinct, but the incidence of EC and GCC is high within these 2 regions [5]. We and others have reported familial aggregation of EC and GCC and increased EC and GCC risk in family members in this high-risk population [6]–[9]. In the Chaoshan high-risk area, the incidence of EC and GCC is not even among population groups, although they are exposed to the similar environment.

**Figure 1:**
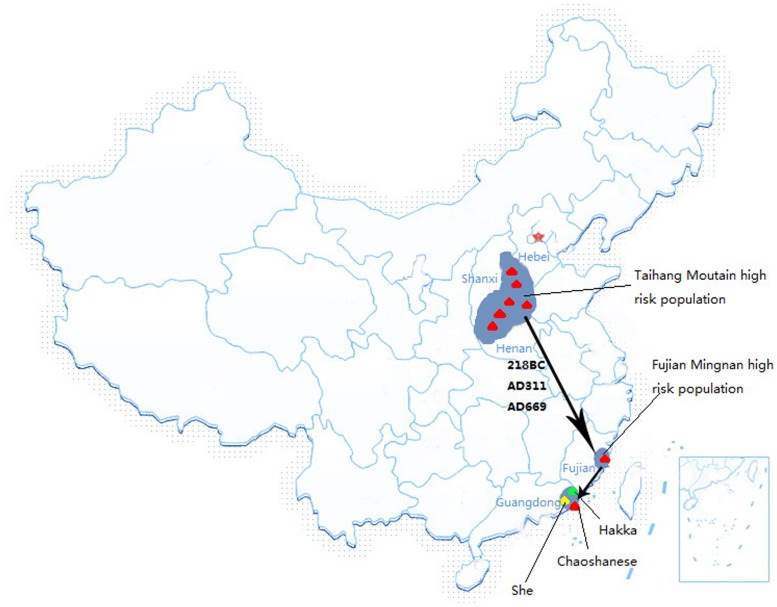
Geographic distribution of the three studied EC and GCC high-risk populations and two low-risk population Hakka and She in Chaoshan area. Arrows show the north-to-south migrations of Han inhabitants from north-central China according to historical records. 218BC, AD311 and AD669 are the three major time periods of north-to-south migrations.

The 3 main populations in Chaoshan area include 2 Han populations – Chaoshanese with Chaoshan dialects and Hakka with Hakka dialects – and one local aboriginal She population. Since the Qing Dynasty (216∼207 BC), the Henan and Shanxi Han people of north-central China migrated into the Chaoshan area in Guangdong province via Fujian province because of war and famine. They gradually became the predominant inhabitants of the Chaoshan area and are called Chaoshanese [10], so the Chaoshan dialect is similar to ancient Chinese. Hakka Chinese originated from the north Han Chinese of the Yellow River and Luohe River basin of the Central Plain. From the Jin Dynasty (266∼316 AD) to the Tong Dynasty (960∼1297 AD), they were forced to move to southern areas also because of wars. When the Hakkas arrived in the Chaoshan area, the Chaoshanese had already settled in the rich plain area, so the Hakkas had to settle in the mountain area, where they lived with the local aborigines, the She population (Fig 1).

The Hakka and Chaoshanese populations show the characteristics of their unique cultures [10]–[13] which have many similarities to northern Han Chinese, including some features of dialect, life style, customs, and habits [10]. The Chaoshan She population is the only aboriginal and minority population. She people mainly work in agriculture, forestry, and animal husbandry; their language and living customs differ from that of the Han population [14]. Although all 3 populations are exposed to a similar geographical environment, only the Chaoshanese have a high incidence of EC and GCC.

Our previous research of Y-chromosome and mtDNA haplogroups concluded that the EC high-risk populations in Taihang Mountain, Fujian Minnan and Guangdong Chaoshan share a similar patrilineal and matrilineal genetic background [15], [16]. In the present study, we further explored the patrilineal genetic structure of EC and GCC patients in Chaoshan high-risk areas and compared it with matched high-risk populations and corresponding low-risk populations. We aimed to examine whether Chaoshan cancer patients have a common ancestry with Taihang Mountain patients and whether they share the same unique Y-chromosome haplotypes. We also compared these data for Y-chromosome single nucleotide polymorphisms (Y-SNPs) and Y-chromosome short tandem repeat (Y-STRs) with that of other Chinese populations from public databases to explore the relative genetic affinity of the studied populations. We first analyzed non-recombining portion of the Y chromosome (NRY) in these 6 populations with 16 East Asian-specific biallelic markers [17], [18] (SNPs), which were characterized by low mutation rate and low probabilities of back and parallel mutation and suitable for tracing early demographic events in human history. Then we investigated the genetic distance among EC and GCC patients with Y-STR loci with relatively high mutation rate and appropriate for analyzing the relationship among close groups and their microevolution [15], [16]. Both Y-SNP and Y-STR analysis results support that the Chaoshan patients have close genetic relatedness with Taihang Mountain patients and the patients have closer relationship with each other than with the high risk population.

## Results

### Distribution of NRY Haplogroups in the 6 Studied Populations in China

Y-SNP genotyping revealed the haplogroup frequencies of the Chaoshan EC or GCC patients, Taihang Mountain EC or GCC patients, and Chaoshan Hakka and She populations. The highest haplogroup of Chaoshan patients was O3a3c1-M117, which is the characteristic haplogroup for Northern East Asians (Table 1). It was also high for Taihang Mountain patients but was significantly lower for Chaoshan Hakka and She populations than Chaoshan patients (p<0.05). Both Chaoshan Hakka and She populations showed a high frequceny of O1a*, the characteristic haplogroup for Southeastern Asians. It was significantly higher for Chaoshan Hakka than Chaoshan patients (p<0.05). The She population showed a unique high frequency of O3a3b* as compared with other studied populations, except the Chaoshan GCC patients, with very low frequency of 2.08%.

**Table 1:** Y-chromosome single nucleotide polymorphism (Y-SNP) haplogroup frequencies of the 6 studied populations (%).

Halplogroup	Chaoshan	Chaoshan	Taihang	Taihang	Chaoshan	Chaoshan
	EC	CC	Mountain EC	Mountain CC	Hakkas (%)	She (%)
	Patients (%)	patients (%)	patients (%)	patients (%)	n = 32	n = 24
	n = 72	n = 48	n = 49	n = 63		
C*	0	0	16.33	9.52	6.25	0
**D/E(M1)**	0	0	0	1.59	0	0
**D1(M15)**	1.39	0	2.04	0	0	0
F*(M89)	4.17	0	0	0	0	0
K*(M9)	1.39	12.5	0	1.59	0	0
O*(M175)	8.33	10.42	2.04	6.35	0	4.17
O3*(M122)	15.28	18.75	26.53	23.81	31.25	29.17
**O3a1(M121)**	2.78	0	2.04	0	0	0
O3a3c*(M134)	5.56	4.17	16.33	23.81	6.25	0
O3a3c1*(M117)	22.22	37.5	24.49	15.87	3.13	8.33
O3a3b*(M7)	0	2.08	0	0	0	20.83
O1a*(M119)	16.67	14.58	2.04	3.17	43.75	20.83
O2a*(M95)	16.67	0	0	6.35	6.25	4.17
O2a1*(M88,M111)	5.56	0	0	0	3.13	0
P*(M45)	0	0	4.08	4.76	0	0
**Q1a1(M120)**	0	0	4.08	3.17	0	12.5

### Principal Component Analysis Revealed Close Affinity among the 4 Patient Groups

**Figure 2:**
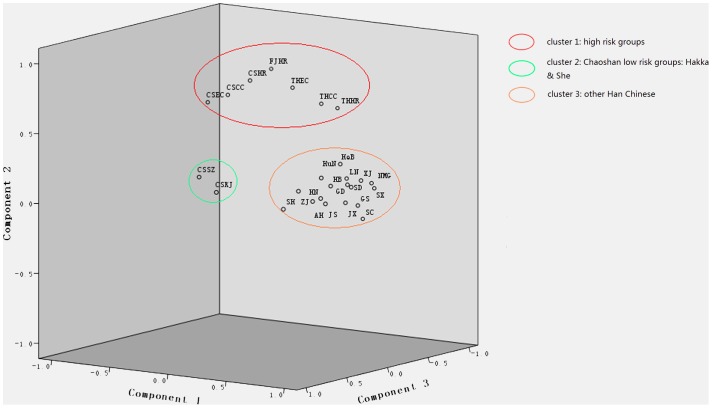
3-D principal component maps of frequencies of Y-chromosome single nucleotide polymorphism (Y-SNP) in Chinese populations. The smaller the distance between populations, the closer the relationship. We divided 26 populations into 3 clusters: 1) Cluster1 (Red circle): 4 patient groups and 3 populations at high risk of esophageal cancer (EC): CSEC: Chaoshan EC patients, CSCC: Chaoshan gastric cardia cancer (GCC) patients, CSHR: Chaoshan high-risk population; FJHR: Fujian high-risk population; THEC: Taihang Mountain EC patients; THCC: Taihang Mountain GCC patients; and THHR: Taihang Mountain high-risk population; 2) Cluster2 (Green circle) Chaoshan Hakka (CSKJ) and She population (CSSZ); 3) Cluster3 (Orange circle) Northern and southern Han populations. Northern Han populations: HeB: Hebei Han; LN: Liaoning Han; XJ: Xinjiang Han; NMG: Neimeng Han; HB: Hubei Han; HN: Henan Han; GS: Gansu Han; SX: Shanxi Han; SD: Shangdong Han. Southern Han populations: GD: Guangzhou Han; SH: Shanghai Han; ZJ: Zhejiang Han; AH: Anhui Han; JS: Jiangsu Han; HuN: Hunan Han; JX: Jiangxi Han; SC: Sichuan Han.

Principal component analysis (PCA) involves a mathematical procedure that transforms a number of correlated variables into a (smaller) number of uncorrelated variables called principal components. The first principal component accounts for as much of the variability in the data as possible, and each succeeding component accounts for as much of the remaining variability as possible. In the principal-component plot, the smaller the distance of two populations, the closer the genetic relationship is between the two. Figure 2 shows the results of principal component analysis, with 3 components (PC1, 2, 3), for Y-SNP frequencies based on genotyping results of the 6 studied populations and additional data for other Chinese Han. For comparison, the haplotype frequencies of 4 high-risk populations from Chaoshan (CSHR), Fujian (FJHR) and Taihang Mountain (THHR) areas were included [15]. The 3 components accounted for 86.2% of the total variation in Y-SNP. The 4 patient groups and 3 high-risk populations clustered together. The Chaoshan She and Hakka populations formed another cluster. The rest of the Northern Han and Southern Han formed another group. The Chaoshan patients and high-risk population were isolated from the Chaoshan Hakka and She populations and Guangzhou Han.

### Positive Correlation between 4 Patient Populations and Chinese Han Populations

Y-SNP haplogroup frequencies for the patient groups and high-risk population from the same area were positively correlated, and frequencies for all patient groups were positively correlated with the Fujian and Chaoshan high-risk populations (Table 2). Frequencies for the Chaoshan EC patients and Chaoshan Hakka were correlated but the coefficient was the lowest. Frequencies for HC were positively correlated with most of the Chinese Han frequencies and those for HNEC were positively correlated with some of the Chinese Han frequencies.

**Table 2:** Correlation analysis of Y-chromosome SNP haplogroup frequencies in the studied populations and 3 high-risk populations and 17 Chinese Han populations.

	Esophageal cancer patients		Gastric cardia cancer patients	
	Chaoshan	Taihang Mountain	Chaoshan	Taihang Mountain
**Taihang Mountain EC**	0.453			
**Chaoshan CC**	0.745**	0.636*		
**Taihang Mountain CC**	0.471	0.897**	0.497	
**Chaoshan high-risk**	0.771**	0.827**	0.828**	0.770**
**Fujian high-risk**	0.618*	0.730**	0.720**	0.614*
**Taihang Mountain high-risk**	0.434	0.691**	0.334	0.830**
**Chaoshan Hakka**	0.550*	0.313	0.362	0.345
**Chaoshan She**	0.411	0.332	0.404	0.287
**Hebei Han**	0.188	0.605*	0.273	0.773**
**Shandong Han**	0.104	0.540*	0.229	0.709**
**Henan Han**	0.122	0.443	0.308	0.522*
**Anhui Han**	0.175	0.505	0.29	0.519*
**Zhejiang Han**	0.35	0.478	0.226	0.625**
**Jiangsu Han**	0.195	0.418	0.264	0.6*
**Shanghai Han**	0.283	0.377	0.343	0.484
**Hubei Han**	0.201	0.515*	0.236	0.727**
**Sichuan Han**	−0.7	0.165	0.057	0.426
**Jixi Han**	0.156	0.303	0.188	0.532*
**Hunan Han**	0.385	0.472	0.263	0.723**
**Gansu Han**	−0.071	0.385	0.08	0.520*
**Liaoning Han**	0.073	0.334	0.133	0.471
**Neimengu Han**	−0.087	0.571*	0.031	0.695**
**Shanxi Han**	−0.005	0.399	0.139	0.638*
**Xingjiang Han**	0.055	0.518*	0.186	0.719**
**Guangdong Han**	0.235	0.449	0.121	0.722**

P<0.01 level (2-tailed).

P<0.05 level (2-tailed).

### Hierarchical Cluster Analysis Isolates Patients and High-risk Population from Other Populations

**Figure 3:**
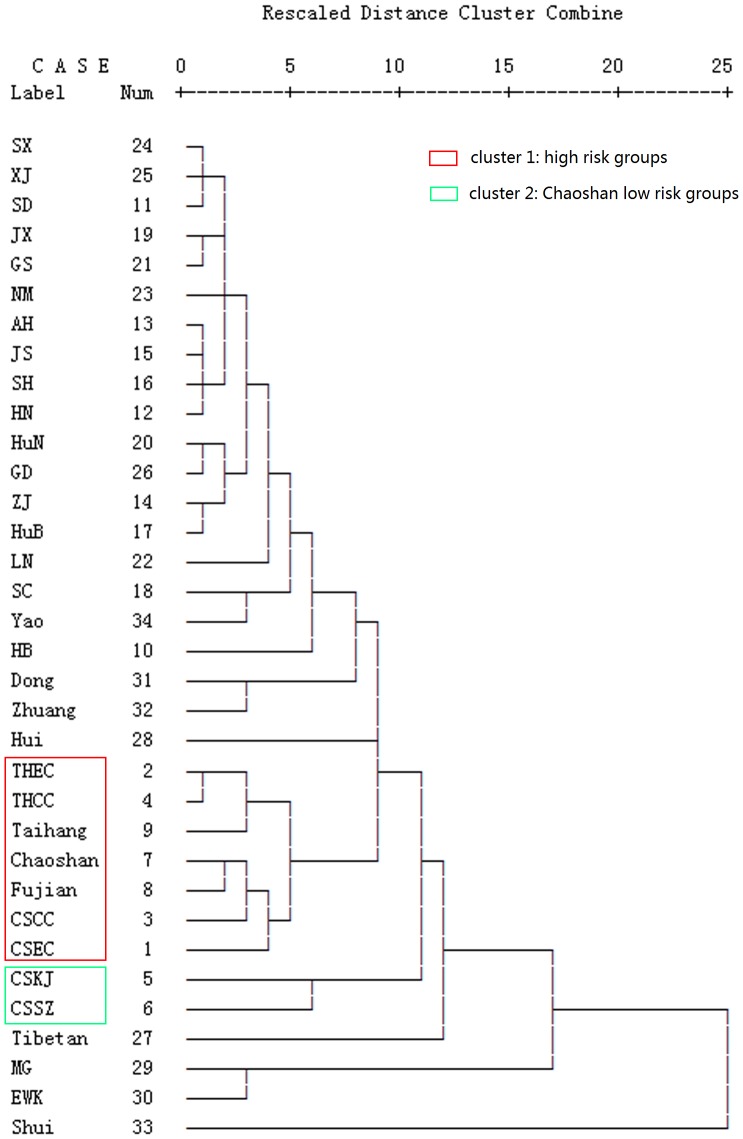
Dendrogram of Y-SNP data. Shows the affinity between the studied populations, the high-risk population, Chinese Han and Chinese minority nationalities. Taihang: Taihang Mountain high-risk population; Chaoshan: Chaoshan high-risk population; Fujian: Fujian high-risk population. The other abbreviations are defined in the Methods and Figure 2.

To study the affinity among the 4 patient groups and their relationship with other Han and minority nationalities, we analyzed Y-SNP data by hierarchical cluster analysis with average linkage (between groups). We compared 17 Chinese Han populations (population information was the same as from principal component analysis), 3 southern minority nationalities (Yao, Zhuang and Dong; [19] and 5 northern minority nationalities (Tibetan, Mongol (MG), Hui, Ewenki (EWK), Shui). The Taihang Mountain patients and high-risk population (Taihang) were genetically close and formed a branch; meanwhile, the Chaoshan patients were genetically close to the Chaoshan and Fujian high-risk populations (Chaoshan, Fujian) and formed another branch (Fig. 3). Then these 2 branches crossed and clustered with Chaoshan Hakka and She populations. All other populations clustered outside the main branch formed by populations from high-risk areas. Therefore, EC or GCC patients and high-risk populations were closer genetically with each other than with Chaoshan Hakka, She and other populations.

### Genetic Distance Analysis and Construction of a Phylogenetic Tree

**Figure 4:**
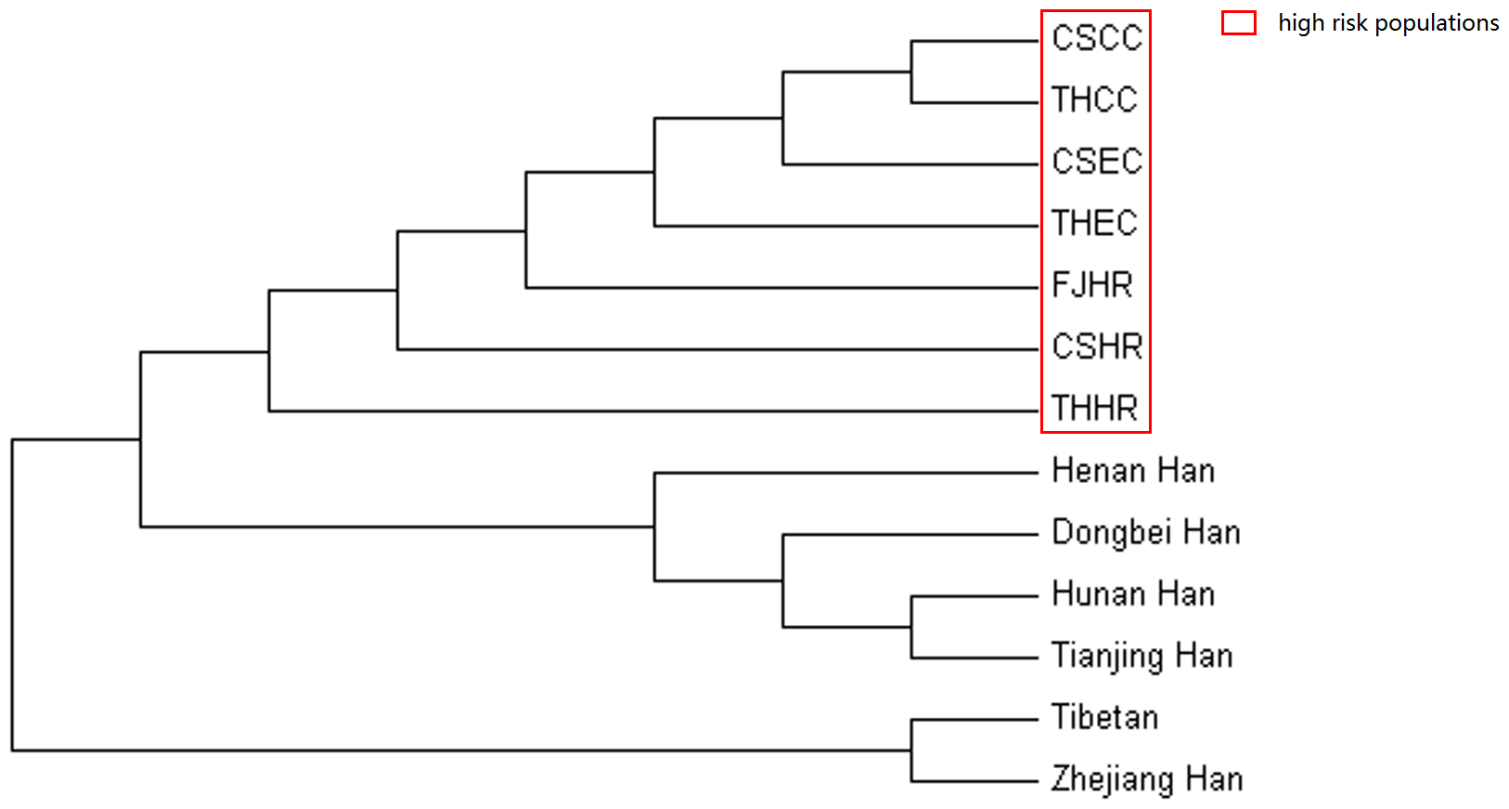
Neighbor-joining tree of genetic distance between patients, high-risk EC population and Chinese Han populations based on Y-chromosome short tandem repeat (Y-STR) data. The 4 patient groups are close to each other and are clustered with the high-risk populations.

We used Y-STR data to investigate the genetic relationships between the 4 patient populations. R_st_ distances between pairs of populations were calculated on the basis of 6 Y-STRs:DYS389 (I, II), DYS390, DYS391, DYS392, DYS393 and DYS394. We included 6 additional Chinese populations and 3 high-risk populations: Zhejiang [20], Henan [21], Dongbei [22], Tianjing [23], Hunan Han [24], and Tibetan [25], and Chaoshan, Fujian, and Taihang Moutain high-risk populations, all of which belong to the Sino-Tibetan language family [15], as do the 4 patient groups. From the R_ST_ distance matrix, we constructed an unrooted neighbor-joining tree (Fig. 4). The patient groups were closer to each other than to the high-risk populations and the other Chinese Han populations.

### Network Analysis of Y-STR Haplogroups of the 4 Patient Groups and 3 High-risk Populations

**Figure 5:**
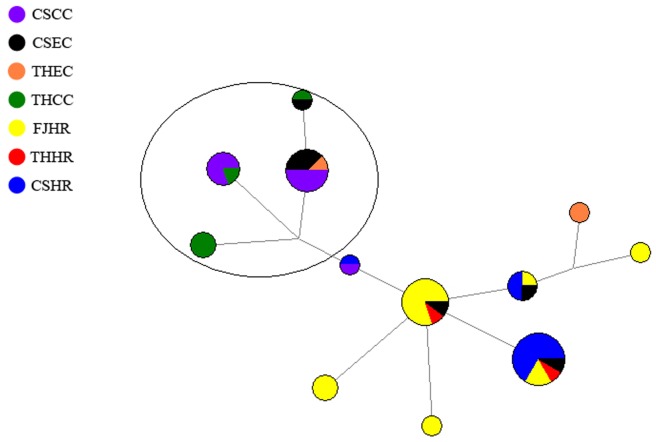
Y-STR network of haplogroup O3a3c1-M117 for patients and high-risk populations belonging to cluster 1 in figure 2. Most patient groups were generated from one node and clustered mainly in one area (circle). Circles represent lineages, area is proportional to frequency, and color indicates population of origin.

The highest haplogroup frequency shared by the Chaoshan patients was O3a3c1-M117 (Table 1). The network for patients and high risk populations was further constructed based on the haplogroup O3a3c1-M117. In all, 12 Henan and 15 Chaoshan EC patients, 17 Chaoshan and 9 Henan GCC patients, and 23 Chaoshan, 8 Henan and 24 Fujian high-risk individuals belonged to haplogroup O3a3c1-M117. Individuals with Y-STR frequency <2 were eliminated from the analysis. Finally, data for 55 individuals were included and analyzed (Fig. 5). The central node was represented by 8 Fujian high-risk individuals, 1 Henan high-risk individual and 1 Chaoshan EC patient. All of the other haplogroup O3a3c1-M117 individuals came from this central node. This central node was connected to 5 one-step neighbors, with 2 neighbors representing 5 Fujian high-risk individuals; the third neighbor represented 8 Chaoshan high-risk individuals, 1 Henan high-risk individual, 2 Fujian high-risk individuals and 1 Chaoshan EC patient; the fourth neighbor represented 2 Chaoshan EC patients, 1 Chaoshan high-risk individual and 1 Fujian high-risk individual; and the fifth neighbor represented 1 Chaoshan GCC patient and 1 Chaoshan high-risk individual. Most patients were generated from the fifth one-step neighbor and thus clustered mainly in one area (circle in Fig. 5). This area included all GCC patients and 5 EC patients, with the remaining 6 EC patients scattered in other nodes.

## Materials and Methods

### Sample Collection and DNA Extraction

Blood samples of 288 unrelated males were collected from the Taihang Mountain and Chaoshan high-risk areas. Informed consent was obtained from all subjects. Subjects were 1) Chaoshan patients–72 EC and 48 GCC patients; 2) Taihang Mountain patients–49 EC and 63 GCC patients; and 3) Chaoshan EC low-risk population–24 She people from Chaoshan Fenghuang Mountain and 32 Chaoshan Hakka from Chaoshan Puning county. Disease in all patients was confirmed pathologically. All participants involved in our study were given written informed consents. The study was approved by the ethical review committee of Shantou University Medical College. Genomic DNA was extracted from whole blood by the TIANamp Blood DNA kit (DP318-03) (Tiangen Biotech Co., Beijing).

### Genotyping of Y-SNPs and Y-STRs

**Figure 6:**
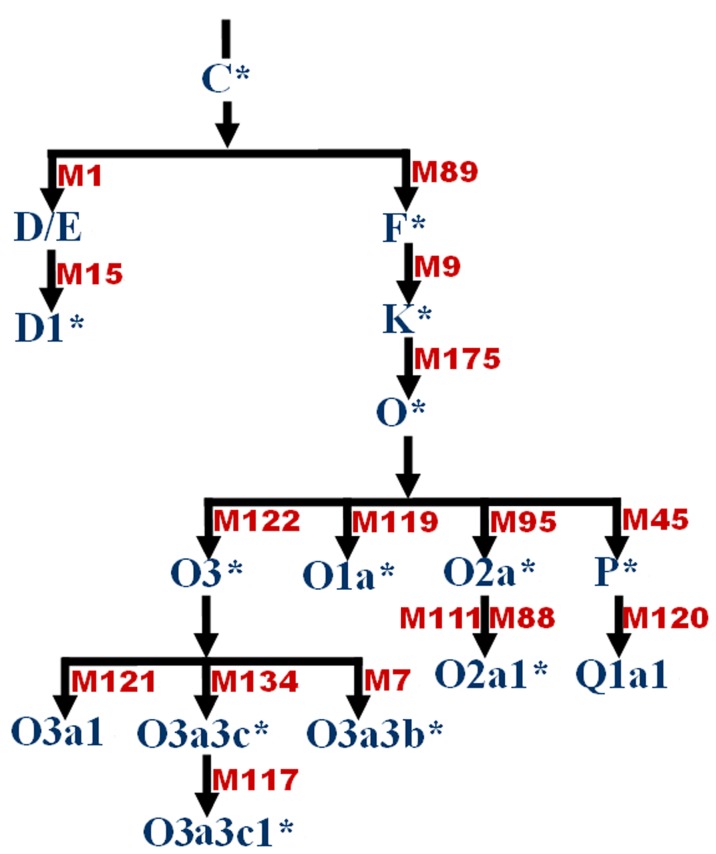
Phylogenetic diagram of 17 haplogroups in Chinese populations based on 16-chromosome biallelic markers drawn according to the non-recombining portion of the Y-chromosome haplogroup tree of East Asia. The most recent markers defining the haplogroups are beside the branches.

Y-SNPs were genotyped by Sequenom MassARRAY iPLEX Gold module (Sequenom Inc.) (PCR primers and extension primers are in Table 3). M1 polymorphism (Alu insertion, also called YAP) was directly analyzed by agarosegel electrophoresis after PCR [26]. STRs were genotyped by fluorescence PCR as previously described [15], and fluorescent-labeled extension products were capillary electrophoresed on an ABI 3730x Genetic Analyzer (ABI, USA). All primers were synthesized by Sangon Co. (Shanghai). In 1999, Su et al. ascertained 17 Y-chromosome haplogroups based on 19 East Asian-specific biallelic markers as the paternal structure of East Asians [19]. The adjusted phylogenetics diagram of Y-SNPs [27] includes nearly 600 SNPs and defines 311 haplogroups. The phylogenetic diagram of 17 haplogroups defined by 16 Y-SNPs is in Figure 6.

**Table 3:** PCR primers and extension primers for 15-SNPs used in Sequenom genotyping.

SNP_ID	WELL	1st-PCRP	2nd-PCRP	UEP_SEQ
**M134**	W1	ACGTTGGATGGAATCATCAAACCCAGAAGG	ACGTTGGATGGGAGAGATACTTTTGATCCC	TTTTGATCCCCACCAAT
**M119**	W1	ACGTTGGATGGGGAGACAGATAATTCTGC	ACGTTGGATGATGGGTTATTCCAATTCAGC	CAATTCAGCATACAGGC
**M88**	W1	ACGTTGGATGCAGTGCTAGAGAGGAAAACC	ACGTTGGATGTATAGGCTATGGCCTAGGTG	TATTCCTGCTTCTTCTGC
**M45**	W1	ACGTTGGATGCAGTAACTCTAGGAGAGAGG	ACGTTGGATGCCTGGACCTCAGAAGGAGC	TCAGAAGGAGCTTTTTGC
**M122**	W1	ACGTTGGATGCAAGGTAGAAAAGCAATTGAG	ACGTTGGATGCTCTGTGTTAGAAAAGATAGC	ccGATTTTCCCCTGAGAGC
**M15**	W1	ACGTTGGATGTGTCCAGAGGGTCTGCTAAC	ACGTTGGATGGGAAGAGTAGAGAAAAGGTG	GAGAAAAGGTGGTACAATG
**M7**	W1	ACGTTGGATGGCATCACCAAAGGGCATGTA	ACGTTGGATGTTGTAGTTGAGTTACTGTT	GTTGAGTTACTGTTCTTCTT
**M95**	W1	ACGTTGGATGTCTCCTAAGCCTACAGGTTG	ACGTTGGATGATGGAGTTCCTGAGGATAAG	GGAAAGACTACCATATTAGTG
**M117**	W1	ACGTTGGATGATTGACAGTTATCAGTTTG	ACGTTGGATGATAACTCACCAAAGGAATGC	CTCACCAAAGGAATGCACATCT
**M111**	W1	ACGTTGGATGGCCAAAAACAACAGAACAAG	ACGTTGGATGTGTGGTACTTGTTTTGTGTG	AGGTAAATTTTGGGGAGAAAAC
**M89**	W1	ACGTTGGATGAAAGGTAGCTGCAACTCAGG	ACGTTGGATGTCCTGGATTCAGCTCTCTTC	CCTAAGGTTATGTACAAAAATCT
**M120**	W1	ACGTTGGATGCGCAATAAAGTATAATTTCCC	ACGTTGGATGAACACACTGCTAATGATCCG	tTCCGTTTTTTGATGTGGAAATA
**M175**	W2	ACGTTGGATGCTACTGATACCTTTGTTTCTG	ACGTTGGATGTGAATCAGGCACATGCCTTC	ATGCCTTCTCACTTCTC
**M9**	W2	ACGTTGGATGCATTGAACGTTTGAACATGTC	ACGTTGGATGCAGAACTGCAAAGAAACGGC	GGCCTAAGATGGTTGAAT
**M121**	W2	ACGTTGGATGCAGCATGATATTTCCACATC	ACGTTGGATGCATCGCTAAACACACGTACC	CACACGTACCATAAATCAAA

### Population and Genotyping

Subjects were genotyped for Y-SNP haplogroup and frequencies were compared among the 4 patient populations and She and Hakka populations (Table S1). Principal component, correlation and hierarchical cluster analyses were used to analyze the relationship among the 6 populations. Three high-risk populations from the Taihang Mountain, Fujian Minnan, and Chaoshan areas and 25 previously published Chinese populations were compared. The 25 Chinese populations were divided into 4 groups by geographic location and nationality [15]: Northern Han (NHs) and northern minority nationalities (NMNs), southern Han (SH) and southern minority nationalities (SMNs).

NH populations were Hebei [28], Liaoning (data provided by the State Key Laboratory of Genetic Engineering and Center for Anthropological Studies, School of Life Sciences, Fudan University), Xinjiang, Gansu, Shanxi, Neimeng, Shandong and Henan [28]; SH populations were Hunan, Hubei, Zhejiang, Jiangxi, Shanghai, Anhui, Jiangsu, Sichuan [28], Guangzhou and Guangxi (data provided by Fudan University); NMN populations were Tibetan, Mongol, Hui, Ewenki, and Shui (data provided by Fudan University); data for 3 southern minority nationalities (Yao, Zhuang and Dong [19] and 5 northern minority nationalities (Tibetan, Mongol, Hui, Ewenki, and Shui populations were provided by Fudan University). Chaoshan patients, Henan patients, Chaoshan Hakka and Chaoshan She population belong to SHs, NHs, SHs, and SMNs, respectively. Guangzhou Han, Chaoshan Hakka, and Chaoshan patients belong to the Guangfu, Hakka, and Fulao/Helao clans, respectively, the 3 major clans in Guangdong Province. Chaoshan She people comprise the major SMNs who live in the Chaoshan area. These 4 populations are geographically proximate.

STRs can be used to analyze minute genetic diversity in close populations, so on the basis of Y-SNP results, Y-STRs were used to analyze the genetic differentiation and origin among patients and high-risk populations (Table S2). We added Y-STR data for 3 high-risk populations from our previous research [15] and for 6 previously published populations: Zhejiang [20], Henan [21], Dongbei [22], Tianjing [23], Hunan Han [24] and Tibetan people [25].

The extent of genetic differentiation of the populations was estimated by the R_st_ statistic on the basis of the Y-STR haplotypes by use of Alrequin 3.1. A neighbor-joining tree was constructed according to the R_st_ distance matrix with use of MEGA 5.1. A network of Y-STR data was constructed by use of Network 4.6.1.1 (www.fluxus-engineering.com). In the network map, individuals with the same mutations of Y-STRs were in the same node, and one node could generate other nodes due to gradual Y-STR mutation [15].

## Discussion

Chaoshanese are descendants of north-central China Han people. North-central Chinese Han began to migrate into southern China beginning in the Qin Dynasty (216 BC). The Han Dynasty (206 BC–220 AD) experienced another 3 waves of large-scale migration into southern China because of the decrease in the native population in this area. Gradually, over 2,000 years, the north-central Chinese Han became the main population – Chaoshanese in the Chaoshan region, called Helao, who directly migrated from north-central China, or Fulao, who first migrated to Fujian Minnan, then to Chaoshan with well-maintained language and customs from north-central China. The Taihang Mountain people in north-central China, Fujian Minnan and Chaoshan areas are well known for their high incidence of EC [15].

With the development of diagnostic techniques and improved epidemiology, more GCC cases have been confirmed in these areas. EC and GCC are the 2 most common cancers in these 3 areas. Our previous genetic research showed that high-risk populations in these 3 areas share a common ancestry [15], [16]. In the present study, we studied Y-chromosome haplogroups of EC and GCC patients from the Chaoshan and Taihang Mountain areas to further explore the paternal genetic background of the patients. We compared the data with 2 low-risk Chaoshan Hakka and She populations and 3 high-risk populations. We first analyzed the distribution of Y-SNP haplogroups among the studied populations. The haplogroup with the highest frequency shared by Chaoshan EC and GCC patients was O3a3c1-M117, one of the northern Han dominant haplogroups, which was also high in Taihang Mountain patients but low in the Chaoshan Hakka and She populations. As compared with Chaoshan patients and the high-risk population, the Chaoshan Hakka and She populations showed a relatively higher frequency of the southern native dominant O1*. Similar to Taihang Mountain patients, Chaoshan patients showed northern Han dominant haplogroups as their highest frequency haplogroups, so Chaoshan and Taihang Mountain patients are relatively closely related.

On Y-SNP principal component analysis, the paternal structure for Chaoshan patients differed from that for Chaoshan Hakka and She populations, although they are in geographic proximity and Chaoshan Hakka are also descendants of north-central Chinese Hans. Chaoshan patients clustered closely with the Fujian and Henan high-risk population and patients, although they are geographically distant. Chaoshan Hakka and She populations clustered together, which agrees with historical records. Chaoshan Hakka mainly inhabit the mountain area, for more gene flow with the She population, who also live in the mountain area. Y-SNP haplotype frequencies were positively correlated among patients, which further supports their close genetic affinity. The results of hierarchical cluster analysis also supported the close genetic affinity among patients and high-risk populations. Phylogenetically, the patient groups were more closely related to each other than with the high-risk population (Fig. 4). Network analysis (Fig. 5) suggested that the patrilineal lineage of haplogroup O3a3c1-M117 individuals was the Taihang Mountain and Fujian high-risk individuals and Chaoshan EC patients, who constituted the central node, and patients of the O3a3c1-M117 individuals from the 2 studied areas were largely from one one-step neighbors containing 1 Chaoshan high-risk individual and 1 Chaoshan GCC patient. The haplogroup O3a3c1-M117 network analysis revealed variation among populations but also a high degree of patient-specific substructure. All 14 GCC patients and 5 of the 11 EC patients fall into one cluster (Fig. 5, circle). Haplogroup O3a3c1-M117 patients may have originated from the same ancestral haplogroup. Thus, we suggest patrilineal genetic affinity among the 2 geographically separated GCC and EC patients in China.

Recent genome-wide association studies from China high-risk areas showed significant association of a variant at 10q23 in PLCE1 and both esophageal squamous cell carcinoma and gastric cardia adenocarcinoma, which highlights the common genetic mechanisms that may contribute to the etiology of both cancers [29]. Though EC and GCC are pathologically distinct, the epidemiology studies [2]–[9], genome-wide association studies and present study all support that EC and GCC may share common genetic structure. EC and GCC are anatomically adjacent and they have similar embryogenesis. They are exposed to similar environmental condition during life. However why they may be affected by a common genetic structure is still unknown.

We suggest that EC and GCC do not occur at random in high-risk populations but are closely associated with a certain patrilineal background structure and these related patients may inherit a pathogenic genetic structure from their common ancestors.

In summary, the patrilineal genetic structure of Chaoshan and Taihang Mountain patients is similar, and patients have closer affinity with each other than with the high-risk populations. The EC and GCC patients share a recent common ancestor. In contrast, the Chaoshan Hakka and She populations have a relatively distant relationship with Chaoshanese people, which may explain in part the high incidence of EC and GCC in Chaoshanese people.

## Supporting Information

Table S1 **Raw data for individual Y-SNP.**
(XLS)

Table S2 **Raw data for individual Y-STR.**
(XLS)

## References

[1] Szumilo J (2009) Epidemiology and risk factors of the esophageal squamous cell carcinoma. Pol Merkur Lekarski 26: 82–85. 19391515

[2] Chen ZF, Dong ZM (2011) Incidence and Geographic Characteristics of Esophagus-gastric Junction Adenocarcinoma. Chinese Journal of Clinical Oncology 38: 57–60.

[3] Tran GD, Sun XD, Abnet CC, Fan JH, Dawsey SM, et al (2005) Prospective study of risk factors for esophageal and gastric cancers in the Linxian general population trial cohort in China. Int J Cancer 113: 456–463. 15455378 10.1002/ijc.20616

[4] Su M, Liu M, Tian DP, Li XY, Zhang GH, et al (2007) Temporal trends of esophageal cancer during 1995–2004 in Nanao Island, an extremely high-risk area in China. Eur J Epidemiol 22: 43–48. 17195051 10.1007/s10654-006-9086-x

[5] Su M, Li XY, Tian DP, Wu MY, Wu XY, et al (2004) Clinicopathologic analysis of esophageal and cardiac cancers and survey of molecular expression on tissue arrays in Chaoshan littoral of China. World J Gastroenterol 10: 2163–2167. 15259058 10.3748/wjg.v10.i15.2163 PMC4724981

[6] Hu N, Dawsey SM, Wu M, Bonney GE, He LJ, et al (1992) Familial aggregation of oesophageal cancer in Yangcheng County, Shanxi Province, China. Int J Epidemiol 21: 877–882. 1468848 10.1093/ije/21.5.877

[7] Chang-Claude J, Becher H, Blettner M, Qiu S, Yang G, et al (1997) Familial aggregation of oesophageal cancer in a high incidence area in China. Int J Epidemiol 26: 1159–1165. 9447394 10.1093/ije/26.6.1159

[8] Hu N, Dawsey SM, Wu M, Taylor PR (1991) Family history of oesophageal cancer in Shanxi Province, China. Eur J Cancer 27: 1336. 10.1016/0277-5379(91)90116-u 1835609

[9] Wang YP, Han XY, Su W, Wang YL, Zhu YW, et al (1993) Esophageal cancer in Shanxi Province, People’s Republic of China: a case-control study in high and moderate risk areas. Cancer Causes Control 3: 107–113. 10.1007/BF00051650 1562700

[10] Wang WZ, Wang CY, Cheng YT, Xu AL, Zhu CL, et al (2010) Tracing the origins of Hakka and Chaoshanese by mitochondrial DNA analysis. Am J Phys Anthropol 141: 124–130. 19591216 10.1002/ajpa.21124

[11] PX (2002) Hakkanese. Chengdu: Chengdu Cartographic Publishing House.

[12] SPH (1999) Guangdong ethnic groups and the regional culture. Guangzhou: Guangdong Higher Education Press.

[13] TH (1997) Headstream of Chaoshan culture. Guangzhou: Guangdong Higher Education Press.

[14] Lin Y, Lai X, Chen G, Xu Y, Huang B, et al (2012) Prevalence and risk factors associated with prehypertension and hypertension in the Chinese She population. Kidney Blood Press Res 35: 305–313. 22377586 10.1159/000336085

[15] Huang H, Su M, Li X, Li H, Tian D, et al (2010) Y-chromosome evidence for common ancestry of three Chinese populations with a high risk of esophageal cancer. PLoS One 5: e11118. 20559544 10.1371/journal.pone.0011118 PMC2886054

[16] Li XY, Su M, Huang HH, Li H, Tian DP, et al (2007) mtDNA evidence: genetic background associated with related populations at high risk for esophageal cancer between Chaoshan and Taihang Mountain areas in China. Genomics 90: 474–481. 17689918 10.1016/j.ygeno.2007.06.006

[17] Jin L, Su B (2000) Natives or immigrants: modern human origin in east Asia. Nat Rev Genet 1: 126–133. 11253652 10.1038/35038565

[18] Li H, Pan WY, Wen B, Yang NN, Jin JZ, et al (2003) Origin of Hakka and Hakkanese: a genetics analysis. Yi Chuan Xue Bao 30: 873–880. 14577381

[19] Su B, Xiao J, Underhill P, Deka R, Zhang W, et al (1999) Y-Chromosome evidence for a northward migration of modern humans into Eastern Asia during the last Ice Age. Am J Hum Genet 65: 1718–1724. 10577926 10.1086/302680 PMC1288383

[20] Wu WW, Zheng XT, Pan LP, Hao HL, Fu T (2005) A study of polymorphisms of 16 Y-STR loci in Han population in Zhejiang. Forensic Sci Technol 5: 11–17.

[21] Feng CJ, Xiang ZD, Shen CB (2005) Polymorphisms of twelve Y-chromosome STR loci in Han population in Henan. Forensic Sci Technol 3: 23–28.

[22] Ba HJ, Ling ZQ, Li S (2007) Polymorphisms of eleven Y-chromosome STR loci in Han population in Northeast of China. J Forensic Med 23: 206–209.

[23] Kuang JZ, Zhu W, Nie TG, Liu Y, Liu MN, et al (2005) Polymorphisms of 12 Y-STR loci in Han population in Tianjin. Forensic Sci Technol 1: 19–26.

[24] Chen SQ, Chen HJ, Zeng XG, Li Q, Zhu ZL, et al (2005) Polymorphism analysis of seven Y-STR loci in Han population in Henan. Chin J Forensic Med 20: 174–176.

[25] Zhao JM, Yuan DY, Kang LL, Liu K, Li SB (2007) Genetic polymorphisms of 14 Y-chromosomal short tandem repeat loci and haplotypes in Tibetan. Chin J Med Genet 24: 94–96. 17285555

[26] Wen B, Shi H, Ren L, Xi HF, Li KY, et al.. (2003) The origin of Mosuo people as revealed by mtDNA and Y chromosome variation. Sci in China (Series C) 33. 10.1360/02yc0207 15382670

[27] Karafet TM, Mendez FL, Meilerman MB, Underhill PA, Zegura SL, et al (2008) New binary polymorphisms reshape and increase resolution of the human Y chromosomal haplogroup tree. Genome Res 18: 830–838. 18385274 10.1101/gr.7172008 PMC2336805

[28] Ke YH, Su B, Xiao JH, Chen H, Huang W, et al (2001) Ychromosome haplotype distribution in Han Chinese populations and modern human origin in East Asians. Sci in China (Series C) 44: 225–232. 10.1007/BF02879329 18726402

[29] Abnet CC, Freedman ND, Hu N, Wang Z, Yu K, et al (2010) A shared susceptibility locus in PLCE1 at 10q23 for gastric adenocarcinoma and esophageal squamous cell carcinoma. Nat Genet 42: 764–767. 20729852 10.1038/ng.649 PMC2947317

